# *Piper* Essential Oils Inhibit *Rhizopus oryzae* Growth, Biofilm Formation, and Rhizopuspepsin Activity

**DOI:** 10.1155/2018/5295619

**Published:** 2018-07-05

**Authors:** Catia A. Almeida, Mariana M. B. Azevedo, Francisco C. M. Chaves, Marcelo Roseo de Oliveira, Igor A. Rodrigues, Humberto R. Bizzo, Paola E. Gama, Daniela S. Alviano, Celuta S. Alviano

**Affiliations:** ^1^Department of General Microbiology, Institute of Microbiology Paulo de Góes, Federal University of Rio de Janeiro (IMPG-UFRJ), CCS Ilha do Fundão, 21941-590 Rio de Janeiro, RJ, Brazil; ^2^EMBRAPA Western Amazon, Rodovia AM 10 km 29, 69010-970 Manaus, AM, Brazil; ^3^School of Farmacy, Department of Natural Products and Food, Federal University of Rio de Janeiro, CCS Ilha do Fundão, 21941-902 Rio de Janeiro, RJ, Brazil; ^4^EMBRAPA Food Technology, Avenida das Américas 29501, 23020-470 Rio de Janeiro, RJ, Brazil

## Abstract

*Piper* is the largest genus of the Piperaceae family. The species of this genus have diverse biological activities and are used in pharmacopeia throughout the world. They are also used in folk medicine for treatment of many diseases in several countries including Brazil, China, India, Jamaica, and Mexico. In Brazil, *Piper* species are distributed throughout the national territory, making this genus a good candidate for biological activity screening. During our studies with *Piper* essential oils, we evaluated its activity against *Rhizopus oryzae*, the main agent of mucormycosis. The main compounds of seven *Piper* essential oils analyzed were *Piper callosum*—safrole (53.8%), *P. aduncum*—dillapiole (76.0%), *P. hispidinervum*—safrole (91.4%), *P. marginatum*—propiopiperone (13.2%), *P. hispidum*—γ-terpinene (30.9%), *P. tuberculatum*—(*E*)-caryophyllene (30.1%), and *Piper* sp.—linalool (14.6%). The minimum inhibitory concentration of *Piper* essential oils against *R. oryzae* ranged from 78.12 to >1250 *μ*g/mL. The best result of total inhibition of biofilm formation was obtained with *Piper* sp. starting from 4.88 *μ*g/mL. Considering the bioactive potential of EOs against planktonic cells and biofilm formation of *R. oryzae* could be of great interest for development of antimicrobials for therapeutic use in treatment of fungal infection.

## 1. Introduction

*Piper* is the largest genus of the Piperaceae family. The species of this genus have diverse biological activities and are used in pharmacopeia throughout the world. They are also used in folk medicine for treatment of many diseases in several countries including Brazil, China, India, Jamaica, and Mexico. In Brazil, *Piper* species are distributed throughout the national territory. Among the aromatic flora of the Amazon region, there are more than a dozen species that provide essential oils that are used by the population for therapeutic purposes. The tea of the decoction of *Piper hispidum* leaves is useful for the treatment of malaria. *Piper marginatum* is used as a tonic, carminative, stimulant, diuretic, and sudorific agent against stomach, liver and gallbladder pain, toothaches, and snake and insect bites [1]. Regasini et al. [2] related trypanocidal activity of the *Piper tuberculatum* extract.

Zygomycosis, also referred to as phycomycosis or mucormycosis, is an aggressive and rapidly progressive infection that primarily occurs in immunocompromised patients. Members of the genera *Rhizopus*, *Mucor*, and *Absidia* are the organisms most commonly isolated from patients with zygomycosis. *Rhizomucor*, *Cunninghamella*, *Apophysomyces*, and *Saksenaea* are other zygomycetes that have been implicated in human diseases. Amphotericin B, as well as its lipid formulation, has been essential for treatment for several decades [3].

The purpose of the present work was to evaluate the anti-*Rhizopus oryzae* activity of *Piper aduncum*, *P. hispidinervum*, *P. callosum*, *P. hispidum*, *P. tuberculatum*, *P. marginatum*, and *Piper* sp. essential oil of leaves.

## 2. Materials and Methods

### 2.1. Plant Material and Essential Oil Extraction

Plant material was obtained from EMBRAPA Experimental Farm, Amazonas, Brazil. A voucher of each specimen was deposited at Federal Agrotechnical School of Machado Herbarium (Table 1). Leaves of *Piper* species were collected between 8 and 9 a.m., dried at room temperature, and coarsely ground into powder just before distillation. The oil was obtained by hydrodistillation in a modified Clevenger apparatus for 5 h [4].

### 2.2. Essential Oil Analyses

Sample of each *Piper* essential oil was analyzed in an Agilent 6890 N gas chromatograph fitted with a 5% diphenyl-95% dimethylpolysiloxane capillary column (DB-5MS, 30 m × 0.25 mm × 0.25 *μ*m). The results were compared to data from the literature [5].

### 2.3. Antifungal Activity Assay

The antifungal activity of *Piper* essential oils was evaluated against *R. oryzae* (UCP1506). The strain used belongs to the culture collection of the “Universidade Católica de Pernambuco,” located in the Nucleus of Research in Environmental Sciences, Catholic University of Pernambuco, Brazil, NPCIAMB/UNICAP. The culture collection is registered in the WFCC.

The microdilution broth method was used according to CLSI reference document M38-A [6] for filamentous fungi. Briefly, the cells were grown in RPMI-MOPS (pH 7.2) for 18 h at 30°C in the presence of different concentrations (1.22 to 1250 *μ*g/mL) of each essential oil. Positive and negative growth controls were performed. Amphotericin B (Sigma) was used as a reference drug, and stock solution was made at 20 mg/mL in sterile distilled water. All experiments were performed in duplicate and repeated twice.

In order to evaluate the fungicide/fungistatic properties of *Piper* essential oils, a 10 *μ*L aliquot was collected from the inhibited cultures and dropped on the surface of potato dextrose agar. The absence or presence of growth in the solid medium was evaluated after 48 h incubation period at 30°C.

### 2.4. Biofilm Formation

The influence of *Piper* essential oils on biofilm formation was determined as described by Singh et al. [7]. Briefly, spores of *R. oryzae* were put in 96-well microtiter plate at 5 × 10^4^ cells per mL in RPMI and treated with twofold serial dilution of each *Piper* essential oil. After incubation for 18 h at 30°C, the culture media was removed and the wells were washed twice with PBS 0.01 M and pH 7.2. Biofilms were stained with 200 *µ*L of 0.1% safranin for 5 min. Then, the supernatants were removed, and the wells were washed twice with PBS. Finally, 200 *µ*L of 30% glacial acetic acid was added to the microplates in order to elute safranine from the matrix. Biofilm formation was estimated by spectrophotometry (SpectraMax M5) at 490 nm.

### 2.5. Red Blood Cell Lysis Assay

The hemolytic activity was evaluated by Franca Rodrigues et al. [8] by mixing 80 *μ*L of a 5% suspension of fresh human red blood cells (O^+^) in PBS with 20 *μ*L of different concentrations of *Piper* sp. essential oil and incubating at 37°C for 1 h. The reaction was slowed by adding 200 *μ*L of PBS, and the suspension was centrifuged (1000 g for 10 min). The supernatant was transferred to a 96-well plate, and cell lysis was quantified by spectrophotometrical measurement of absorbance at 540 nm, as previously described. The maximal lysis and blank control were obtained by replacing the extract sample with an equal volume of PBS or distilled water, respectively.

### 2.6. Rhizopuspepsin Inhibition

In order to evaluate a possible mode of action of the *Piper* essential oils, the inhibition of rhizopuspepsin (Sigma) activity was determined as previously described by Buroker-Kilgore and Wang with some modifications [9]. First, 59 *μ*L of the rhizopuspepsin solution was mixed with 1 *μ*L inhibitor, 20 *μ*L BSA (1 mg/mL), and 20 *μ*L buffer (pH 3.0). After 1 h incubation at 37°C, 100 *μ*L of Bradford solution (0.025% Coomassie Blue G-250, 11.75% ethanol, and 21.25% phosphoric acid) previously diluted (1 : 1) was added. Negative control was performed by adding the substrate immediately after the incubation period. Finally, the plate was read on a spectrophotometer (SpectraMax M5) at 595 nm. One unit of enzyme activity was defined as the total enzyme that causes an increase of 0.001 in unit of absorbance under the conditions of the standard assay. The inhibitors tested were *Piper* essential oils (48 *μ*g/mL) and 10 mM Pepstatin A (standard inhibitor).

### 2.7. Antioxidant Activity of *Piper* spp. Essential Oils

The antioxidant activity was evaluated qualitatively [10, 11] by application of 0.5 *μ*L of each essential oil and 7-hydroxycalamenene (as standard) on a plate of silica gel 60 F_254_ and eluted with hexane-ethyl acetate (9 : 1). The plates were treated with a 0.2% methanolic solution of DPPH and read just after spraying and after 45 min.

## 3. Results and Discussion

The average oil yield obtained was 0.65% (dry wt.). The compounds present in the essential oils from *Piper* species used are shown in Table 2. Quantitative and/or qualitative variations were observed among samples of *Piper*.

The essential oils of *P. aduncum*, *P. hispidinervum*, *P. callosum*, *P. marginatum*, *P. hispidum*, *P. tuberculatum*, and *Piper* sp. were analyzed by GC and GC-MS, and the percentage of identified components is given in Table 2.

The major compounds of *P. aduncum* and *P. hispidinervum* were identified as dillapiole (76%) and safrole (91.4%), respectively. In the oil of *P. callosum*, the main components were safrole (53.8%) and *α*-pinene (12.2%). Major components of *P. marginatum* were propiopiperone (13.2%) and *δ*-3-carene (11.3%). *P. hispidum* presented the terpinene isoforms *γ*-terpinene (30.9%) and *α*-terpinene (14.4%) as main compounds. *β*-Pinene (15%) and caryophyllene oxide (13.3%) were the major constituents of *P. tuberculatum*, while the sesquiterpenes linalool (14.6%) and nerolidol (13.8%) were identified in the *Piper* sp. oil.

Dillapiole has been described as acaricidal (*Rhipicephalus* (*Boophilus*) *microplus*), larvicidal and insecticidal (*Anopheles marajoara*, *Aedes aegypti*, and *Solenopsis saevissima*), and antifungal (*Aspergillus fumigatus*) agent. Safrole demonstrated antileishmanial (*L. major*, *L. mexicana*, *L. braziliensis*, and *L. donovani*) activity. Propiopiperone exhibited antifungal activity against *Cladosporium cladosporioides* and *C. sphareospermum*. Oyedemi et al. [12] showed the activity of *γ*-terpinene against *Proteus vulgaris* and *Escherichia coli*. Our group previously described [13] the activity of (+)-*β*-pinene against *Cryptococcus neoformans* and *Candida albicans*. Other promising activity described by our group [14] was linalool-rich essential oil of *Lippia alba* against two dermatophytes *Trichophyton rubrum* and *Epidermophyton floccosum* [12–21].

The results of the MIC assay of *Piper* essential oils and amphotericin B against *R. oryzae* are shown in Table 3.

Sartoratto et al. [22] considered MIC values between 50 and 500 *µ*g/mL as strong activity, MIC values between 600 and 1500 *µ*g/mL as moderate activity, and above 1500 *µ*g/mL as weak activity [21]. According to this classification, it could be stated that *Piper* sp., *P. marginatum*, and *P. hispidum* essential oils present high activity, *P. tuberculatum* and *P*. *hispidinervum* present moderate activity, and *P*. *aduncum* and *P*. *callosum* against *R. oryzae* planktonic cells present weak activity.

**Figure 1:**
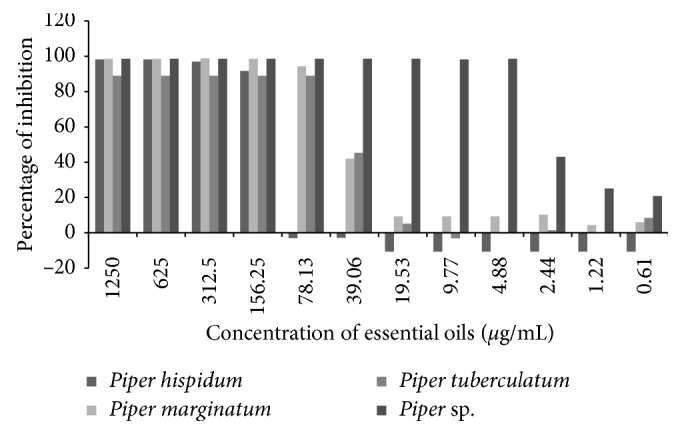
Effect of *Piper* essential oils against *R. oryzae* biofilm formation. The plates were incubated at 30°C for 18 h.

Based on previous MIC results, the essential oils tested on biofilm formation were *P. hispidum*, *P. marginatum*, and *P. tuberculatum*; *Piper* sp. *Rhizopus oryzae* biofilm formation in the presence of each *Piper* essential oil was inhibited in lower concentration than MIC for all species tested (Figure 1).

In their natural environments, most of bacteria and fungi change from a planktonic to a sessile state forming the so-called biofilms. Biofilms are sessile microbial and fungal communities that are strongly attached to surfaces and to each other; in such phase, they are protected by a polymeric extracellular matrix (ECM), constituted primarily of polysaccharides. According to Singh et al. [7], the major compounds of biofilm matrix are GlcN and GlcNAc. The cell wall of zygomicetes is also mainly formed by GlcN and GlcNAc polymer constituents of chitosan and chitin, respectively. Then, our results on MIC and inhibition of biofilm formation could be associated with each other. The essential oil of *Piper* sp. showed the most active agent against the two cell forms, planktonic and biofilm [7, 23].

**Figure 2:**
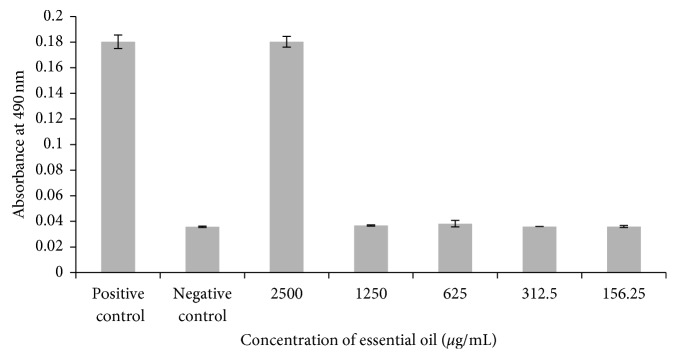
Hemolytic assay after treatment with various concentrations of *Piper* sp. essential oil.

*Piper* sp. essential oil was the most active agent against planktonic cells and biofilm formation (78.12 and 4.88 *μ*g/mL, resp.). However, this essential oil displayed hemolytic activity (Figure 2) at higher concentration (2500 *μ*g/mL), making it a promising antifungal candidate.

**Figure 3:**
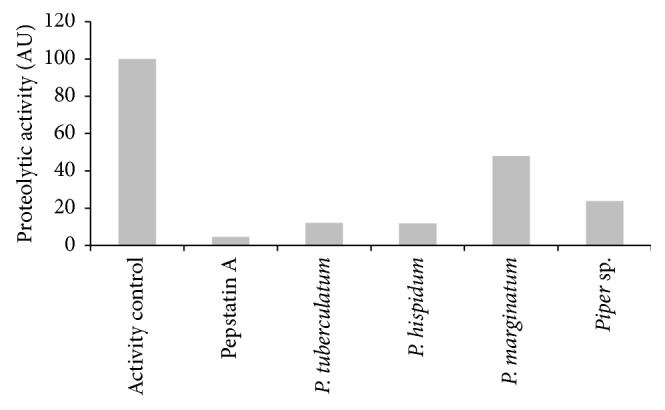
Proteolytic activity of rhizopuspepsin after overnight treatment with 48 *µ*g/ml of *Piper* essential oils. The plates were incubated at 37°C.

Other important mechanism of action is the inhibition of rhizopuspepsin and/or saps, a class of enzymes secreted for *R. oryzae* and other *Rhizopus* species [24]. The results in Figure 3 showed inhibition of proteolytic activity of rhizopuspepsin when *Piper* essential oils were used, mainly *P. hispidum* and *P. tuberculatum* which inhibited 11.8% and 12.05% of enzymatic activity, respectively.

The antioxidant activity was evaluated after TLC of *Piper* essential oils. It was not possible to identify regions containing substances with activity even after 45 min of application of DPPH. Terpenes are the most significant class of compounds present in essential oils. Among them, several monoterpene hydrocarbons, oxygenated monoterpenes, and sesquiterpenes are often reported as weak antioxidant agents [25]. However, due to the complexity of essential oils' composition, some antioxidant activity was expected. Thus, further investigation will be necessary in order to evaluate other antioxidant methods.

## 4. Conclusion

This study showed the promising anti-*Rhizopus oryzae* activity of *Piper tuberculatum*, *P. hispidum*, and *Piper* sp. against planktonic cells, biofilm formation, and rhizopuspepsin which makes these essential oils useful in formulating strategies to limit the growth of *R. oryzae*.

## Figures and Tables

**Table 1:** Deposit number and deposit location of plant material.

Plant material	Deposit number	Deposit location	Name of herbarium
*Piper aduncum*	10,480	INPA^1^	INPA herbarium
*Piper tuberculatum*	6,797	IFAM^2^	EAFM herbarium
*Piper hispidum*	6,796	IFAM	EAFM herbarium
*Piper marginatum*	6,798	IFAM	EAFM herbarium
*Piper callosum*	6,794	IFAM	EAFM herbarium
*Piper hispidinervum*		IFAM	EAFM herbarium
*Piper* sp.		IFAM	EAFM herbarium

^1^National Institute of Amazonas Research; ^2^Federal Institute of Amazonas.

**Table 2:** Main components from *Piper* spp. essential oils.

				Area (%)						
Peak	LRI calc	LRI lit	Identification	*P. callosum*	*P. aduncum*	*P. hispidinervum*	*P. marginatum*	*P. hispidum*	*P. tuberculatum*	*Piper* sp.
1	924	924	*α*-Thujene	0.1			0.1	0.1		
2	931	932	*α*-Pinene	12.2	1.5	0.1	2.0	1.3	9.4	
3	946	946	Canphene	0.4	0.1			0.1		
4	971	969	Sabinene	3.0			0.1			
5	975	974	*β*-Pinene	7.7	3.5		1.5	1.1	15.0	
6	985	981	6-Methyl-5-hepten-2-one					0.6		
7	989	990	Myrcene	0.6		0.1	1.3	1.2		
8	1004	1002	*α*-Phellandrene	0.1			1.5	0.5		
9	1009	1008	*δ*-3-Carene			0.2	11.3	0.4		
10	1014	1014	*α*-Terpinene	0.7			0.2	14.4		
11	1022	1022	*p*-Cimene	0.3	0.3	0.3	0.3	12.1		
12	1025	1024	Limonene	0.7	0.4	0.2			1.6	
13	1026	1025	*β*-Phellandrene				1.0	1.4		
14	1028	1026	1,8-Cineole	3.7						
15	1034	1032	(*Z*)-*β*-Ocimene		0.4	0.3	6.0			
16	1044	1044	(*E*)-*β*-Ocimene		0.8	0.7	8.3			
17	1055	1054	*γ*-Terpinene	1.8			0.3	30.9		
18	1085	1086	Terpinolene	0.5		1.2	0.3	7.3		
19	1098	1098	Linalool	0.3	0.2		1.1			14.6
20	1134	1135	*trans*-Pinocarveol		0.2					
21	1152	1155	Isoborneol	0.1						
22	1173	1174	Terpinen-4-ol	0.7				1.0		
23	1182	1179	*p*-8-Cymenol			1.0				
24	1187	1186	*α*-Terpineol	0.5	0.1					
25	1193	1194	Myrtenol		0.1					
26	1314	1285	Safrole	53.8		91.4	4.6			
27	1332	1335	*δ*-Elemene				0.3			
28	1370	1374	*α*-Copaene	0.5	0.5		4.8	0.5	1.3	
29	1379	1387	*β*-Bourbonene				0.9			
30	1385	1387	*β*-Cubebene				0.3			
31	1387	1389	*β*-Elemene				0.6		3.0	
32	1402	1403	Methyl eugenol	7.6			5.4			
33	1413	1417	(*E*)-Caryophyllene	0.7	6.0	0.3	6.3	5.3	30.1	14.4
34	1423	1430	*β*-Copaene				0.3		2.8	
35	1438	1439	Aromadendrene					1.4		
36	1447	1452	*α*-Humulene	0.1	0.9		0.7	0.4		7.1
37	1450	—	n.i.				0.3			
38	1456	1457	Croweacin				0.9			
39	1468	1471	4,5-Di-epi-aristolochene					0.3		
40	1475	1476	*β*-Chamigrene					1.6		
41	1480	1489	*β*-Selinene				1.7	8.1	2.6	5.5
42	1489	1498	*α*-Selinene					9.0	1.7	5.0
43	1499	1500	Epizonarene					0.1		
44	1471	1478	*γ*-Muurolene	0.4						1.6
45	1474	1484	Germacrene D	1.0	0.6		2.9			
46	1488	1493	*Epi*-cubebol		0.4					
47	1490	1494	Bicyclogermacrene		0.5	1.0	3.9			
48	1491	1494	Sarisan			0.3				
49	1495	1500	Pentadecane		0.3	0.2				
50	1498	1505	Germacrene A				0.2			
51	1500	1500	*α*-Muurolene					0.2		
52	1500	1506	*β*-Bisabolene						9.1	
53	1509	1514	Cubebol		0.8					
54	1510	—	n.i.				1.6			
55	1518	1517	Myristicin		2.4	2.0				
56	1513	1513	*γ*-Cadinene					0.4		3.5
57	1516	1520	7-*epi*-α-Selinene					0.2		
58	1501	1511	*δ*-Amorphene		0.3					
59	1518	1522	*δ*-Cadinene	0.4			0.8	1.1		1.2
60	1530	1545	Propiopiperone				13.2			1.2
61	1544	1548	Elemol				1.1			
62	1554	1555	Elemicin	1.4			2.7			
63	1559	1561	(*E*)-Nerolidol		0.5		1.0		6.5	13.8
64	1571	1577	Spathulenol		0.5	0.7	4.1		2.2	2.5
65	1576	1582	Caryophyllene oxide		1.5		1.8		13.3	10.1
66	1580	1601	*α*-Cedrol							3.1
67	1582	1590	Globulol					1.2		
68	1584	1592	Viridiflorol		1.2					
69	1593	1624	Selina-6-en-4-ol							7.3
70	1606	—	n.i.					0.6		
71	1618	—	n.i.					0.1		
72	1625	1620	Dillapiole		76.0					
73	1625	1627	1-*epi*-Cubenol				0.9			
74	1631	1642	2-Hydroxy-3,4-methylenedioxypropiophenone				1.0			
75	1637	1638	*epi*-*α*-Cadinol					0.5		
76	1640	1644	*α*-Muurolol	0.2						
77	1648	1649	*β*-Eudesmol	0.2			0.9			
78	1651	1658	Selin-11-en-4*α*-ol					2.0		
79	1652	1652	*α*-cadinol				1.2		1.5	3.0
80	1655	1658	*neo*-Intermedeol				0.5			

**Table 3:** MIC values (*μ*g/ml) of *Piper* essential oils and amphotericin B against *R. oryzae*.

Essential oil	MIC	MFC
*Piper aduncum*	>1250	ND
*Piper hispidinervum*	1250	ND
*Piper callosum*	>1250	ND
*Piper marginatum*	156.25	>1250
*Piper hispidum*	312.5	>1250
*Piper tuberculatum*	625	>1250
*Piper* sp.	78.12	>156.5
Amphotericin B	0.98	1.95
Posaconazole	1.56	1.56

MIC: minimal inhibition concentration; MFC: minimal fungicide concentration; ND: not determined.
